# Modeling Hybridization Kinetics of Gene Probes in a DNA Biochip Using FEMLAB

**DOI:** 10.3390/microarrays6020009

**Published:** 2017-05-29

**Authors:** Ahsan Munir, Hassan Waseem, Maggie R. Williams, Robert D. Stedtfeld, Erdogan Gulari, James M. Tiedje, Syed A. Hashsham

**Affiliations:** 1 Department of Civil and Environmental Engineering, Michigan State University, East Lansing, MI 48823, USA; ahsan.muneer@gmail.com (A.M.); hasan_wasim@hotmail.com (H.W.); kronlei2@msu.edu (M.R.W.); stedtfel@msu.edu (R.D.S.); 2 Department of Chemical Engineering, University of Michigan, Ann Arbor, MI 48109, USA; gulari@umich.edu; 3 Center for Microbial Ecology, Michigan State University, East Lansing, MI 48823, USA; tiedjej@msu.edu; 4 Department of Plant, Soil, and Microbial Sciences, Michigan State University, East Lansing, MI 48823, USA

**Keywords:** hybridization kinetics, microfluidics, DNA biochips, vancomycin resistance genes, FEMLAB

## Abstract

Microfluidic DNA biochips capable of detecting specific DNA sequences are useful in medical diagnostics, drug discovery, food safety monitoring and agriculture. They are used as miniaturized platforms for analysis of nucleic acids-based biomarkers. Binding kinetics between immobilized single stranded DNA on the surface and its complementary strand present in the sample are of interest. To achieve optimal sensitivity with minimum sample size and rapid hybridization, ability to predict the kinetics of hybridization based on the thermodynamic characteristics of the probe is crucial. In this study, a computer aided numerical model for the design and optimization of a flow-through biochip was developed using a finite element technique packaged software tool (FEMLAB; package included in COMSOL Multiphysics) to simulate the transport of DNA through a microfluidic chamber to the reaction surface. The model accounts for fluid flow, convection and diffusion in the channel and on the reaction surface. Concentration, association rate constant, dissociation rate constant, recirculation flow rate, and temperature were key parameters affecting the rate of hybridization. The model predicted the kinetic profile and signal intensities of eighteen 20-mer probes targeting vancomycin resistance genes (VRGs). Predicted signal intensities and hybridization kinetics strongly correlated with experimental data in the biochip (*R*^2^ = 0.8131).

## 1. Introduction

Microfluidic biochips are widely used for high-throughput analysis of mutations, expression profiles, and in identification of microorganisms [1] and are based on highly selective solid-phase nucleic acid hybridization [2]. The sensitivity of these biochips strongly depends on hybridization kinetics, temperature of hybridization, target concentration, surface probe concentration, washing protocol, and other factors [3,4,5]. The role and optimal selection of these parameters is critical for developing efficient hybridization protocols for microfluidic biochips [6,7]. Numerous experimental investigations and theoretical models have been reported for describing the kinetics and thermodynamics of solid-phase nucleic acid hybridization [8,9,10]. However, besides the pioneering work reported on computer simulations of reaction–diffusion kinetics [11,12] providing a sound theoretical understanding of these phenomenon, modeling and experimental validation of DNA:DNA hybridization in biochips is generally lacking. 

In this work, a three-dimensional mathematical model for selective nucleic acid hybridization in a microfluidic biochip was developed followed by comparison of theoretical predictions with experimental data. The biochip used was developed by Xeotron, now owned by Invitrogen (Carlsbad, CA, USA) and has been previously described [13]. To model and simulate hybridization kinetic curves, a finite element software package (FEMLAB, a package for COMSOL Multiphysics; COMSOL, Inc., Burlington, MA, USA) was used. The model accounts for fluid flow, convection and diffusion in the channel as well as diffusion on the reaction surface. The model was used to predict hybridization kinetics of eighteen 20-mer probes targeting vancomycin resistant genes. This gene was chosen as a model because emergence of antibiotic resistance associated with pathogenic bacteria is an alarming global issue [14,15] and numerous hybridization-based biochip platforms are being developed to diagnose multi-drug resistant bacteria [16,17,18,19,20]. Effect of target concentration, temperature, recirculation flow rate and rate constants on the hybridization kinetics, with target concentration, rate constants, and temperature being the most important parameters affecting the rate of hybridization were investigated. Recirculation flow rate was found to play a vital role in determining whether the hybridization system was transport- or reaction-limited. The predictive capability and performance of the model and its potential for application in optimizing hybridization protocols and chip geometry were considered adequate with some exceptions.

## 2. Materials and Methods 

A three-dimensional mathematical model was implemented to describe the hybridization of a DNA target to a surface-immobilized probe in a glass wafer microfluidic biochip (obtained from Invitrogen, formerly Xeotron, [13]). The microfluidic biochip had several independent hybridization wells (15 μm deep and 56 μm in diameter) where probes were attached and connected through several channels according to light-directed synthesis technology previously reported [13,21]. The utility of this biochip for microarrays including the related validation experiments (e.g., materials used, probe attachment parameters, etc.) was previously studied and published [13,21]. 

**Figure 1:**
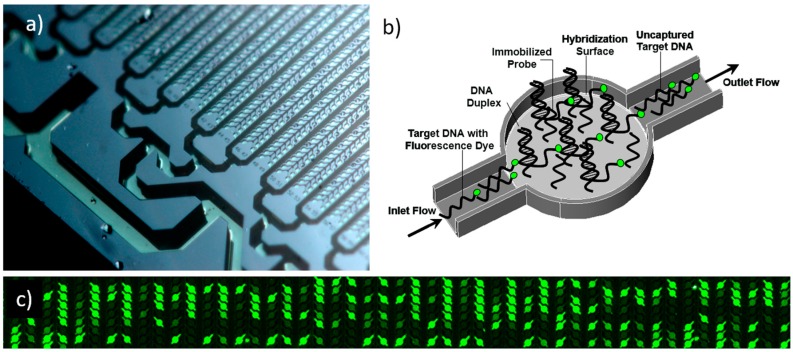
Macroscopic image of the microfluidic biochip used in this study for nucleic acid hybridization (**a**), the three-dimensional geometry of a single micro-reactor with a hybridization surface (**b**), and a section of the DNA biochip showing various micro-channels with hybridization signals of various intensities (**c**). The channel was 14 μm wide and 15 μm deep. The center cylindrical portion had a diameter of 56 μm. (**a**) Photo credit Kurt Stepnitz, Michigan State University, property of MSU Board of Trustees, used with permission.

In this study, to evaluate the hybridization kinetics, only a single hybridization well was considered (Figure 1). In physical experiments, the target DNA is captured in many micro-reactors available for hybridization. Though the effect of ion concentration in the hybridization buffer is known to significantly impact hybridization, we have left the hybridization buffer constant according to published methods as this has been previously optimized [22].

The target DNA solution flows into the micro channel at a constant flow rate. The flow of an aqueous solution of DNA inside a small channel is assumed as uniform. The target DNA molecules are transported by convection along the flow direction where they are free to diffuse. Fluid flow is circulated through the chip multiple times, so the fluid flow rate evaluated in this study is, in fact, the recirculation flow rate. When the target DNA molecule reaches the hybridization well, the well-known association–dissociation reaction occurs between the target DNA and the immobilized probes. Both the association and dissociation events are modeled here using a differential kinetic equation. 

### 2.1. Mathematical Model

Only one of the many hybridization wells, connected with a microchannel, is considered in the three-dimensional model (Figure 1). The fluid containing target DNA flows from left to right where the incoming flow profile is characterized by fully developed laminar flow, i.e., it is parabolic with zero velocity at the channel walls. Fluid flow in the channel follows the Navier–Stokes equation,

(1) $$\rho \frac{\partial u}{\partial t}-\eta \nabla^{2}u+\rho \left(u\cdot \nabla \right)u+\nabla p=F$$

(2) ∇⋅u=0

where u denotes the velocity field vector, ρ is density, η is the dynamic viscosity, and p is pressure. At steady-state, the first time-dependent term disappears. The external force, F, such as gravity can be neglected in such miniaturized systems. 

The incoming flow has a small concentration of the target DNA which is described by a convection–diffusion equation of the form,

(3) $$\frac{\partial c}{\partial t}+u\cdot \nabla c=D\nabla^{2}c$$

where c denotes the concentration of solution-phase targets, u is the identical velocity field vector given in Equation (1). The diffusion coefficient, D, was estimated using Table 1 of Chan et al. [23], where it was reported that the diffusion coefficient of DNA depends on its length and decreases with increasing base pairs [23].

Once the target DNA reaches the bottom of the hybridization well, the association and dissociation kinetics are described by using an ordinary differential equation,

(4) $$\frac{dB}{dt}=k_{on}c(R_{t}-B)-k_{off}B$$

where *k_on_* represents the association rate constant, *k_off_* is the dissociation rate constant, *R_t_* is the total surface concentration of probes, and *B* is the surface concentration of bound targets at time, *t*. The association rate constant, *k_on_*, depends on the DNA sequence involved, the temperature, and the ionic strength of the medium but not to the point that it would drastically affect the order of magnitude. Based on the experimental results reported in literature [11,24,25], it was assumed that the association rate constant does not change appreciably over the temperature range used in our simulation (25–55 °C). Therefore, a value of 10^6^ M^−1^s^−1^ for *k_on_* was used in these simulations. The dissociation rate constant, *k_off_*, was calculated using the thermodynamic model presented in literature [12]. In this model, standard Gibbs free energy and hybridization energy for different DNA sequences was used to calculate the dissociation rate constant at a specific temperature. The model also assumes that the DNA targets do not diffuse on the surface and that there is no leakage of the molecules at the edges of the surface.

### 2.2. Numerical Simulations

Numerical simulations of a single hybridization well were performed with COMSOL Multiphysics (FEMLAB), a finite element software package, which uses two geometries and three application modes to solve the formula. The first geometry is three-dimensional and represents the channel connecting the micro well. Within this geometry, the model used two application modes: incompressible Navier–Stokes, and convection and diffusion to model the transport of the target DNA. The second geometry is two-dimensional and simulates the reaction surface with the diffusion application mode. It is assumed that the hybridization reactions take place on a 2D surface (bottom surface of the micro-wells) whereas bulk flow of incoming target DNA is resolved on a 3D geometry. To correctly model the multi-scale physics, extrusion-coupling variables were defined. These variables couple the mass transport from 3D bulk flow using a surface boundary condition to the hybridization reactions on a 2D surface. In COMSOL Version 4.2 and later, instead of using extrusion-coupling variables, a Surface Reaction interface can be used with a single 3D geometry. This interface resolves surface coverage of the adsorbed species, with a mass source or sink automatically coupled to the surface boundary condition in the bulk mass transport equation. The meshing around the channel was not greater than 10 μm and around the hybridization well it was smaller than 0.2 μm. In all simulations, an “insulation” boundary condition was chosen for the non-reactive surfaces at the top and the bottom of the microchannel except where the hybridization occurred. The model is solved in two steps using different solvers. First, it solves the incompressible Navier–Stokes application mode with a non-linear solver followed by the time-dependent solver to simultaneously solve the convection and diffusion and the diffusion application modes. The model predicts the concentration of duplexes formed due to interactions between the target DNA and immobilized probes inside the hybridization well.

**Figure 2:**
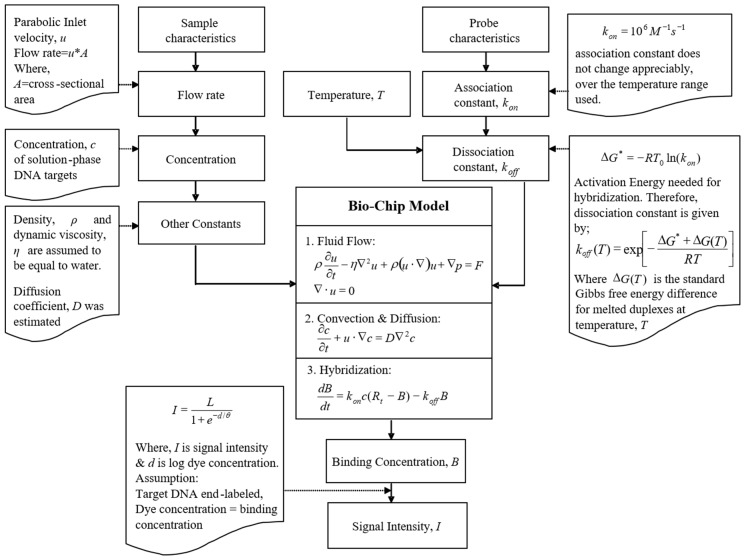
Schematic of data flow in the modeling framework.

In order to correlate the duplex concentration obtained through our model with the hybridization signal intensities, we used Equation (5) [26],

(5) $$I=\frac{L}{1+e^{-d/\theta }}$$

where I is log fluorescence intensity, *d* is log dye concentration, θ defines the spread and slope of the linear range of the curve and the “background” level (set to 1.0 for Cy3 dye), *L* is the upper limit of the dynamic range (set to 3.0). Preset values were taken from the study [26]. For our simulation, we assumed that there was only one dye molecule attached per molecule of target DNA because it was end-labeled. The complete algorithm and the data flow used for our model are illustrated in Figure 2.

### 2.3. Experimental Validation

The mathematical model was validated using the real-time hybridization experiments performed on Xeotron hybridization station (Xeotron Corp., Houston, TX, USA—now part of Invitrogen). Eighteen targets were designed to complement the 18 probes for the hybridization experiments (Table 1). However, this is not a large enough sample size to make predictions about the effect of individual melting temperatures on hybridization kinetics due to other confounding factors such as mismatches [27]. The targets (45–52 mers) were synthesized and amine modified on the 3′ end by Operon Biotechnologies Inc. (Huntsville, AL, USA - part of MWG-Biotech AG, Ebersberg, Germany). The targets were coupled with Cy3 (Amersham Biosciences, Little Chalfont, United Kingdom, Cat. No. PA23001) and then dissolved in dimethyl sulphoxide (Sigma-Aldrich, St. Louis, MO, USA, Cat. No. D2438 in 75 mM sodium carbonate buffer at pH 9.0). A hybridization buffer containing 35% deionized formamide (Thermo-Fisher Scientific, Waltham, MA, USA), 6x SSPE (pH 6.6; Invitrogen; 1x SSPE contains 0.18 M NaCl, 10 mM NaH_2_PO_4_, and 1 mM EDTA pH 7.7), and 0.4% Triton X-100 (Sigma-Aldrich) [22]. Several hybridization experiments were performed to assess the effects of temperature, concentration and flow rate. The real-time data images of nucleic acid hybridization were obtained using OSA Reader v1.91 software (IMSTAR, Paris, France) in conjunction with a CCD camera (resolution 10 μm per pixel) attached to a Nikon Eclipse 50i fluorescent microscope (100× magnification; Nikon Instruments, Melville, NY, USA).

## 3. Results and Discussion

### 3.1. Theroretical Analysis

**Figure 3:**
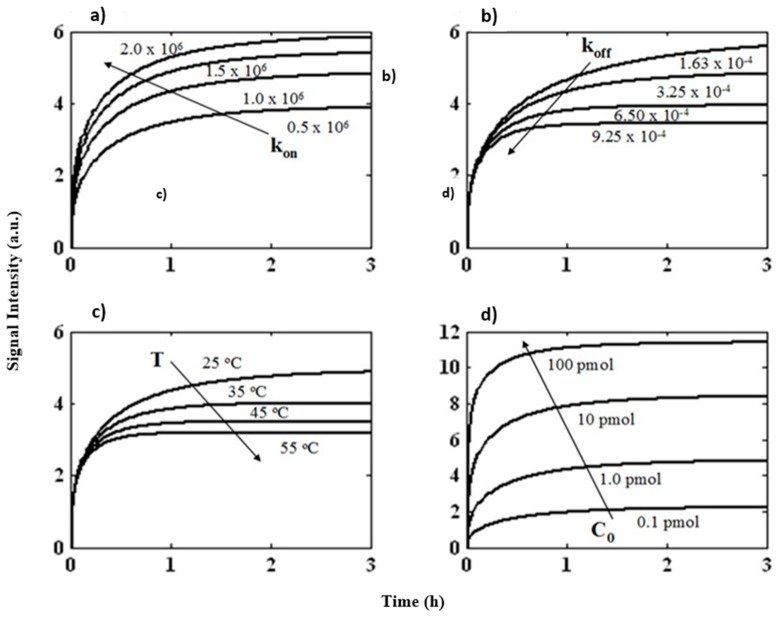
Influence of different variables on the hybridization kinetics of vancomycin resistance genes. (**a**) Increase in hybridization with increase in association constant *k_on_*, (**b**) decrease in hybridization with increase in dissociation constant *k_off_*, (**c**) decrease in hybridization with increase in temperature (T), and (**d**) increase in hybridization with increase in initial target concentration (C_0_). In each case where one parameter is varied, the rest remain constant.

The quantitative prediction of the effect of operating parameters on the signal intensity of DNA hybridization is investigated by performing 28 different simulations. A variety of parameters have distinct influences on the hybridization kinetics of *VanC* (5′-GCCTTATGTATGAACAAATGGCT-3′) type gene (Figure 3). The binding constant plays an important role in estimating the maximum DNA duplex concentration at equilibrium [28]. The key factors which give a DNA molecule high or low affinity are the association rate constant, *k_on_*, and dissociation rate constant, *k_off_*. The influence of these parameters was investigated by changing *k_on_* from 0.5 × 10^6^ M^−1^s^−1^ to 2.0 × 10^6^ M^−1^s^−1^ while keeping *k_off_* unchanged and vice versa with *k_off_* ranging from 1.63 × 10^−4^ s^−1^ to 9.25 × 10^−4^ s^−1^. The model showed that the signal intensity increased with increasing forward rate constant, which is expected because of the rapid rate of probe–target association (Figure 3a). In accordance, the signal intensity decreased with increasing dissociation rate constant which was expected because duplexes will dissociate at a faster rate than they are formed (Figure 3b). Simulations were also performed to examine the effect of temperature on binding mechanism and it was observed that with increasing temperature, the signal intensity decreased (Figure 3c) primarily because DNA:DNA duplexes become unstable at higher temperatures, thereby resulting in dissociation [29]. One of the most important parameters for optimizing the assay is the concentration of target DNA [30]. In order to investigate this effect on hybridization kinetics, simulations were completed with the initial DNA target concentration (C_0_) ranging from 0.1 pmol to 100 pmol. As shown in Figure 3d, we observed high signal intensities at a higher concentration of target DNA. This is due to higher duplex formation between the target DNA and the probes available at the capture site.

**Figure 4:**
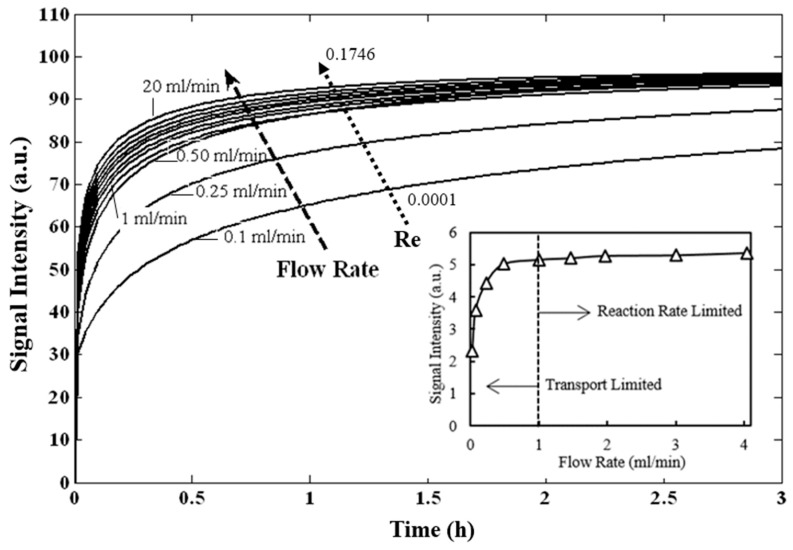
Influence of flow rate and Reynold’s number on the hybridization kinetics of vancomycin resistance genes. Insert: Signal intensity and flow rate at 14 h hybridization.

Flow rate is one of the key parameters that determines whether the kinetics of DNA hybridization in the microfluidic biochip is transport-limited or reaction-limited [31]. Here, the term “flow rate” actually describes the recirculating flow rate. The capture of target DNA is transport-limited if the DNA supply is lower than the demand from the hybridization reaction. In contrast, the capture of target DNA is reaction-limited if the number of DNA molecules binding to the probe per unit time remains similar for different flow rates. To investigate this effect, simulations were carried out by varying the flow rate from 0.1 mL/min to 20 mL/min in the model (Figure 4). We observed that the signal intensity increased appreciably with an increase in flow rate from 0.1 mL/min to 1 mL/min but remained almost the same for flow rates above 1 mL/min. A flow rate of 4 mL/min does not seem to enhance the kinetics more than that observed at a flow rate of 1 mL/min which suggests that the system has become reaction-limited and there is more supply of target DNA than the demand from the hybridization reaction. Therefore, increasing the flow rate in this region will not be an effective way to increase signal intensity. At lower flow rates (<1 mL/min) the hybridization reaction becomes transport-limited because lower volumes of target DNA solution are available at the capture area per unit of time and fewer molecules have a chance to be captured [32]. It has also been suggested that forced convention may drive more biomolecules into an area of detection [33]. Therefore, longer incubation times are necessary to reach an equivalent signal intensity to the reaction-limited case. These results demonstrate that flow rate is a key parameter controlling the binding mechanism. Thus, the developed model enabled determination of optimum flow rate for faster and more sensitive measurements. 

For each of these flow rates, dimensionless parameters including the Reynold’s number and Péclet number have been calculated (Table S1).

### 3.2. Prediction of Hybridization Kinetic Curves

**Figure 5:**
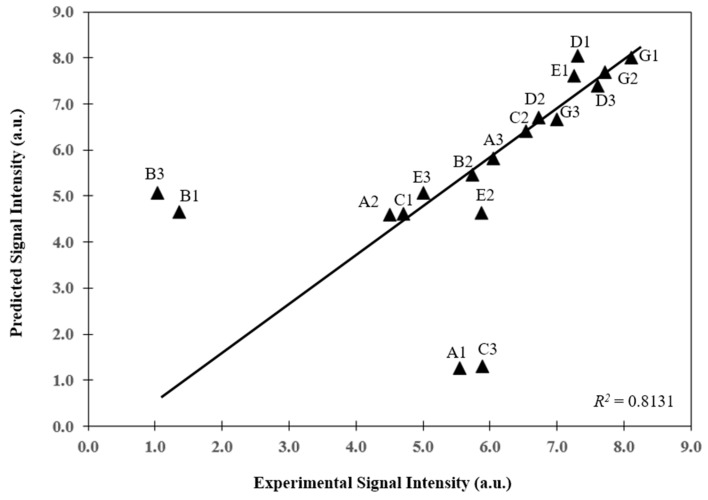
Predicted signal intensity of vancomycin resistance genes compared to experimental signal intensity after 14 h of hybridization (*R*^2^ = 0.8131). Other parameters (Re, Pe) according to Table 2.

The developed mathematical model was used to predict the hybridization kinetic profiles for eighteen 20 mer vancomycin resistance genes (VRGs) probes (Table 1). The predicted values were compared with the experimental work done on same biochip under similar conditions. Dissociation rate constant, *k_off_*, was calculated for each gene using thermodynamic model [12] whereas the association rate constant, *k_on_*, was kept constant throughout the simulations. Other parameters were obtained from literature as described in previous section and were kept constant in the simulations. A comparison of the model predictions of signal intensities after 14 h of hybridization with the experimental signal intensities obtained on the same biochip under similar conditions showed that the model could predict the final signal intensities reasonably well (*R*^2^ = 0.8131) (Figure 5). It was also able to predict the hybridization kinetic profiles for most of the genes but in few cases, the model overestimated or underestimated the final signal intensities. 

**Figure 6:**
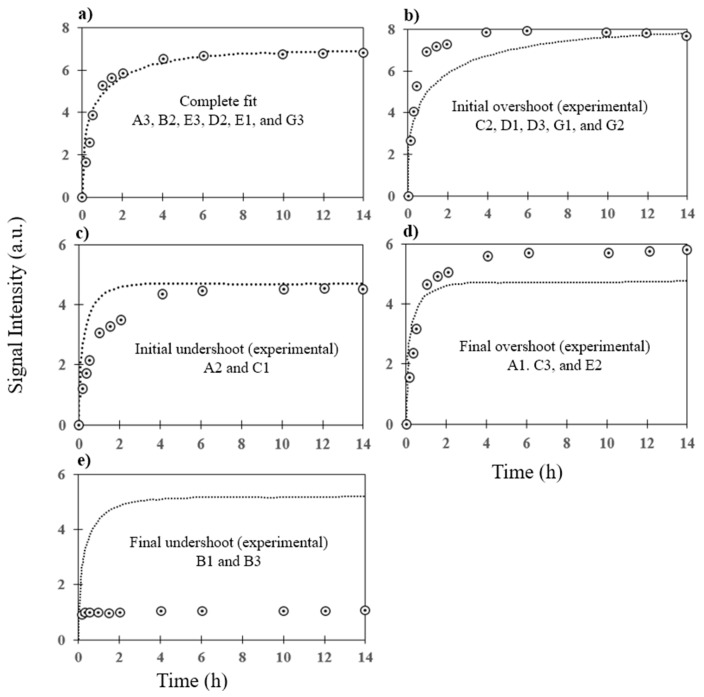
Association curves for hybridization for probes exhibiting (**a**) complete fit (A3, B2, E3, D2, E1, and G3), (**b**) initial overshoot (C2, D1, D3, G1, and G2), (**c**) initial undershoot (A2, C1), (**d**) final overshoot (A1, C3, and E2), and (**e**) final undershoot (B1 and B3). Solid lines represent the predicted signal intensities whereas circles represent the experimental results obtained on the same biochip.

The 14-h association curve and signal intensity was accurately predicted for six of the VRGs probes using the developed model (Figure 6a). The model failed to accurately predict the hybridization curves of six other probes but successfully estimated the signal intensities. We observed that the model overestimated the trend which may be due to the poor estimation of binding constants (C2, D1, D3, G1, G2; Figure 6b) whereas it underestimated the hybridization kinetics (A2 and C1; Figure 6c). For six probes, the model failed to predict final signal intensity and association curves (A1, C3, E2, B1, and B3; Figure 6d). We also observed that in most of the cases, the association of target DNA with the immobilized probes monotonically increased until reaching a plateau. However, for few cases, the signal intensities were initially high before plateauing (D3, G1; Figure 6b). This overshoot in signal intensities might have been caused by the interaction of multiple target molecules with a given probe. Multiple targets bound to a given probe in meta-stable state are either desorbed or displaced by another target to reach a stable state, thereby resulting in late lower signal intensities. Similar overshoot behavior has been observed in the adsorption of polymer molecules [34]. This phenomenon is not considered in the model, and may not exactly predict probe association behaviors. While these outliers could be caused by probe characteristics such as melting temperature, due to the small sample size used here and the large variability observed with probes [27], it is impossible to make definitive claims without evaluating a larger number of probes as these outliers could also be caused by other factors such as mismatches [27]. 

We also observed, in predicted results and in hybridization experiments, that the signal intensities of most of the selected vancomycin gene targets did not change much after 6 h of hybridization, therefore shorter time (ca. 6 h) could have been chosen for hybridization instead of 14 h. In this way, the model can be used to estimate the optimized time for hybridization experiments, thereby enhancing hybridization experiments and in some cases reducing the overall detection time. 

## 4. Conclusions

The developed three-dimensional mathematical model identified sample concentration, flow rate, binding constants, and temperature as crucial parameters affecting the rate of DNA hybridization for VRGs. The flow rate is the most important parameter that determines whether the kinetics of DNA hybridization in the microfluidic biochip is transport or reaction-limited. Based on comparison between the predicted and experimental results on the DNA hybridization for VRGs, it was observed that the model predicts hybridization of DNA in the biochips except for a few cases where it either overestimates or underestimates the results. It also predicted that shorter time could have been chosen for our DNA hybridization experiments. Overall, the model provided an understanding of complex DNA:DNA hybridization kinetics in microfluidic biochips. It could play a vital role in optimizing hybridization protocols for antibiotic resistance genes and the development of chip and chamber geometries for detecting them, which is very important in point-of-care testing where multiple targets need to be detected simultaneously using low sample concentrations.

## Figures and Tables

**Table 1:** Relevant hybridization parameters of 18 probes targeting vancomycin resistance genes.

Probe ID	Probe Sequence	ΔG ^a^	GC ^b^	Tm ^c^
vanA-1	ATCTTTCGTATTCATCAGGAAG	−14.3	36.4%	61.8
vanA-2	GAAAAAGGCTCTGAAAACGCAGTT	−18.5	41.7%	62.8
vanA-3	CGTTATCCCCGTATGATGGC	−16.2	55.0%	60.9
vanB-1	ATGAATAGAATAAAAGTCGCAA	−13.3	27.3%	52.3
vanB-2	GGCTGCGATATTCAAAGCTCCG	−18.7	54.5%	65.8
vanB-3	TATATCGGGTGCTTGGATGCAGAGG	−21.1	52.0%	67.7
vanC-1	GCCTTATGTATGAACAAATGGCT	−16.4	39.1%	58.1
vanC-2	GATGGCTGTATCCAAGGACTGCTTG	−20.4	52.0%	65.8
vanC-3	CCTCAAAAGGGATCACTAAAGTCAC	−18.0	44.0%	60.2
vanD-1	TTATATTGGAATCACAAAATCCGGCG	−18.9	38.5%	66.1
vanD-2	GAGGCCGTTACCGGGAGTGGGTAGG	−24.3	68.0%	72.3
vanD-3	TTTTTTAAGATTCATCAGGAACACAGCCGGA	−25.1	38.7%	70.5
vanE-1	AGGGACAAGACCTACAAAAAGTCGA	−19.3	44.0%	62.2
vanE-2	CCGAATGAGGCAGGCTCATCAAAAG	−20.9	52.0%	69.3
vanE-3	GGAATTAGCAAGGTAGAACGAAAAA	−17.1	36.0%	59.9
vanG-1	TACCAGGCTTTACCTCGCACAGTCG	−22.4	56.0%	68.0
vanG-2	AAAGCTCTGGGCTGTTCGGGTTTTT	−21.5	48.0%	68.3
vanG-3	ATTGGTCTATCGTTCTCCCAAATGT	−18.4	40.0%	61.8

^a^ Gibbs free energy (ΔG) expressed in kcal/mol; ^b^ GC content; ^c^ Melting temperature (Tm) expressed as °C.

**Table 2:** Dimensionless parameters (Reynold’s number (Re) and Péclet Number (Pe)) calculated for each flow rate.

Flowrate (mL/min)	0.01	0.1	0.25	0.5	1	2	20
Velocity (m/s)	6.03 × 10^−6^	6.03 × 10^−5^	1.51 × 10^−4^	3.01 × 10^−4^	6.03 × 10^−4^	1.21 × 10^−3^	1.21 × 10^−2^
Re	0.0001	0.0009	0.0022	0.0044	0.0087	0.0175	0.1746
Pe	1.16	11.63	29.07	58.15	116.30	232.60	2325.95

When the Pe > 1, the effects of convection exceed those of diffusion in determining the overall mass flux.

## Supplementary Materials

The following are available online at www.mdpi.com/2076-3905/6/2/9/s1, Table S1: Calculation of dimensionless parameters including the Reynold’s number and Péclet number.
